# Prevalence and Specificity of Chemoreceptor Profiles in Plant-Associated Bacteria

**DOI:** 10.1128/mSystems.00951-21

**Published:** 2021-09-21

**Authors:** Claudia Sanchis-López, Jean Paul Cerna-Vargas, Saray Santamaría-Hernando, Cayo Ramos, Tino Krell, Pablo Rodríguez-Palenzuela, Emilia López-Solanilla, Jaime Huerta-Cepas, José J. Rodríguez-Herva

**Affiliations:** a Centro de Biotecnología y Genómica de Plantas (CBGP), Universidad Politécnica de Madrid (UPM) - Instituto Nacional de Investigación y Tecnología Agraria y Alimentaria (INIA), Madrid, Spain; b Área de Genética, Facultad de Ciencias, Instituto de Hortofruticultura Subtropical y Mediterránea La Mayora, Universidad de Málaga-Consejo Superior de Investigaciones Científicas (IHSM-UMA-CSIC), Málaga, Spain; c Department of Environmental Protection, Estación Experimental del Zaidín, Consejo Superior de Investigaciones Científicas, Granada, Spain; d Departamento de Biotecnología-Biología Vegetal, Escuela Técnica Superior de Ingeniería Agronómica, Alimentaria y de Biosistemas, Universidad Politécnica de Madrid (UPM), Madrid, Spain; Wageningen University

**Keywords:** MCP, chemoreceptor, chemotaxis, methyl-accepting chemotaxis protein, plant-associated bacteria

## Abstract

Chemosensory pathways are among the most abundant prokaryotic signal transduction systems, allowing bacteria to sense and respond to environmental stimuli. Signaling is typically initiated by the binding of specific molecules to the ligand binding domain (LBD) of chemoreceptor proteins (CRs). Although CRs play a central role in plant-microbiome interactions such as colonization and infection, little is known about their phylogenetic and ecological specificity. Here, we analyzed 82,277 CR sequences from 11,806 representative microbial species covering the whole prokaryotic phylogeny, and we classified them according to their LBD type using a *de novo* homology clustering method. Through phylogenomic analysis, we identified hundreds of LBDs that are found predominantly in plant-associated bacteria, including several LBDs specific to phytopathogens and plant symbionts. Functional annotation of our catalogue showed that many of the LBD clusters identified might constitute unknown types of LBDs. Moreover, we found that the taxonomic distribution of most LBD types that are specific to plant-associated bacteria is only partially explained by phylogeny, suggesting that lifestyle and niche adaptation are important factors in their selection. Finally, our results show that the profile of LBD types in a given genome is related to the lifestyle specialization, with plant symbionts and phytopathogens showing the highest number of niche-specific LBDs. The LBD catalogue and information on how to profile novel genomes are available at https://github.com/compgenomicslab/CRs.

**IMPORTANCE** Considering the enormous variety of LBDs at sensor proteins, an important question resides in establishing the forces that have driven their evolution and selection. We present here the first clear demonstration that environmental factors play an important role in the selection and evolution of LBDs. We were able to demonstrate the existence of LBD families that are highly enriched in plant-associated bacteria but show a wide phylogenetic spread. These findings offer a number of research opportunities in the field of single transduction, such as the exploration of similar relationships in chemoreceptors of bacteria with a different lifestyle, like those inhabiting or infecting the human intestine. Similarly, our results raise the question whether similar LBD types might be shared by members of different sensor protein families. Lastly, we provide a comprehensive catalogue of CRs classified by their LBD region that includes a large number of putative new LBD types.

## INTRODUCTION

To ensure cell survival, bacteria have to adapt to changing environmental conditions (1). For this, bacterial cells are equipped with an array of different signal transduction systems that sense different environmental stimuli, such as osmolarity, oxygen tension, temperature, pH, light, nutrients, toxins, and other chemicals (2). Chemosensory pathways represent one of the primary bacterial signal transduction mechanisms, and more than half of all the bacterial genomes contain signaling genes (3). Most chemosensory pathways appear to mediate chemotaxis (3), whereas others have been associated with type IV pilus-based motility (4) or alternative cellular functions such as the control of second messenger levels (4, 5).

In a canonical chemosensory pathway, signals are perceived by binding specific molecules to the ligand binding domain (LBD) of chemoreceptors (CRs), which modulates the activity of the CheA autokinase and the subsequent transphosphorylation to the CheY response regulator. In canonical CRs, the extracytosolic LBD is flanked by two transmembrane (TM) regions, a cytosolic HAMP domain, and a signaling domain (MCPsignal). While the CR signaling domain (MCPsignal) is highly conserved, LBDs are rapidly evolving domains (6), which reflects the wide variety of chemoeffectors to be sensed. To date, more than 80 different LBD families have been identified (7, 8), and new types of LBDs continue to be discovered (9). The thermodynamic parameters for ligand binding to the individual CRs are very similar to those for binding to specific LBDs (10, 11), supporting the idea that the molecular determinants for signal recognition by CRs are located in the LBD. Further evidence of this came from the construction of chimeric receptors recombining LBDs with other signaling domains (e.g., autokinase domains), where the LBD was proved to define the function of the chimera (12, 13). Thus, while the conserved MCPsignal domain can be used to identify CRs, their LBDs allow them to be classified on the basis of their function (7, 8).

On the other hand, there is evidence suggesting that the genomic repertory of CRs is related to bacterial lifestyle (14, 15). For instance, it has been shown that plant-associated bacteria (PAB) possess a particularly large number of CRs (8, 16), indicating that chemosensory signaling is indeed an important requisite for plant-bacterium interactions. This is of particular relevance for plant pathogens and symbionts, for which it has been shown that flagellum-mediated chemotaxis is required for optimal virulence or symbiosis establishment (17–25). Plants represent complex habitats for colonization by different kinds of microorganisms, and PAB species can colonize the plant rhizosphere, phyllosphere, or endosphere (26). Motile sensory behavior has been shown to play a key role in the establishment of plant-microbe interactions, since bacteria that can sense and rapidly navigate toward niches optimal for growth and survival will have a clear competitive advantage (27–29). These considerations are valid for both pathogenic and nonpathogenic relationships between microorganisms and plants (8, 16). Similarly, microbial inhabitants of the phyllosphere, comprising the aerial part of plants, have to deal with the challenges of life on leaf surfaces, where flagellar motility confers advantages in terms of epiphytic fitness (30). The epiphytic lifestyle also represents the initial stage of foliar colonization by many bacterial phytopathogens, preceding entry into the leaf apoplast via wounds or natural plant openings (e.g., stomata) (30). However, despite their biological significance, the function and cognate signal have been determined for only a limited number of CRs from PAB, and very little information exists on their phylogenetic and ecological specificity.

In order to study those LBD types most tightly coupled to the plant-associated lifestyle, here we comprehensively identified the CR genes in all known bacterial lineages and classified them according to their LBDs, with a particular focus on the LBD types linked to a plant-associated lifestyle. As such, we employed a novel *de novo* methodology to extract putative LBD regions from all CR sequences and group them into homology-based clusters (i.e., putative LBD types). This analysis allowed us to identify hundreds of LBD types highly specific for PAB species, many of them unknown. We further found that the taxonomic distribution of the majority of PAB-specific LBD clusters is only partially explained by phylogeny, suggesting that niche and host adaptation might have played relevant roles for their selection. Together, these results form a solid basis for the design of experiments aimed at identifying CRs that are essential for plant-microbe interactions and virulence.

## RESULTS

### Towards a global catalogue of chemoreceptors in plant-associated bacteria.

**FIG 1:**
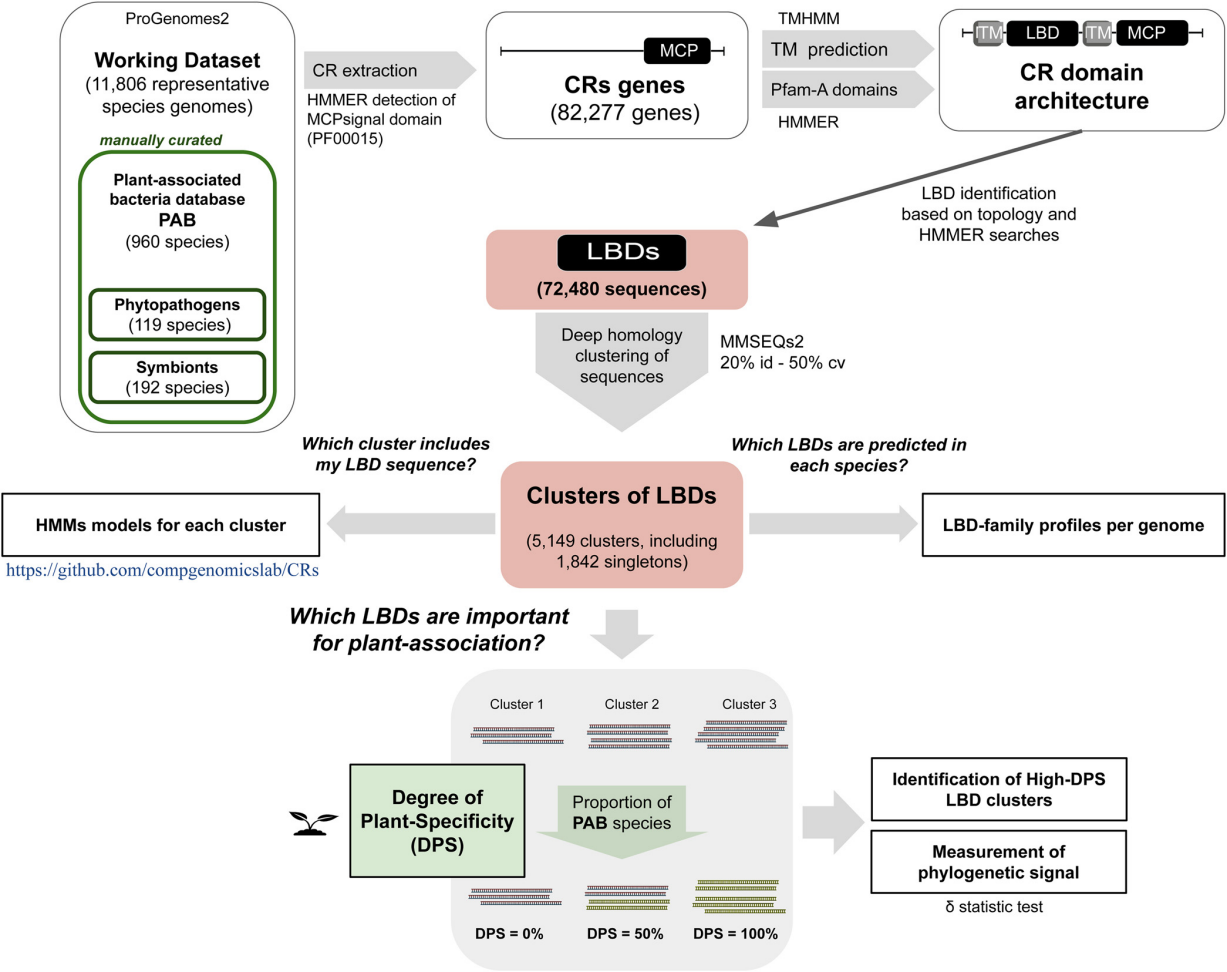
Schematic view of the bioinformatics pipeline used to identify CRs that are potentially relevant for plant association. From a set of 11,806 representative prokaryotic genomes, 82,277 protein sequences were mined using HMM-based searches against the MCPsignal Pfam domain (PF00015). CR topology was analyzed by predicting transmembrane regions (TMs) and Pfam domains. Based on the topological analysis, LBD regions were predicted and a set of 72,480 LBD sequences was obtained. Clustering of LBDs based on sequence homology (20% minimum sequence identity with at least 50% sequence coverage) resulted in 5,149 clusters or subfamilies of LBDs, of which 1,842 contained a single sequence. To study a possible link between the LBD profiles and plant-associated lifestyle, a manually curated subset of 960 representative species of plant-associated bacteria (PAB) was generated, including phytopathogen (119) and symbiont (192) subsets. The determination of the proportion of PAB LBDs present in each cluster allowed us to assign the degree of plant specificity (DPS) value for each LBD subfamily. Subsequent analysis of high-DPS clusters identified LBD clusters that are potentially important for bacterium-plant associations. Furthermore, the validation of the high-DPS clusters as good ecological indicators was corroborated by measuring their phylogenetic signal. A detailed step-by-step description of the process can be found in Materials and Methods.

In order to maximize the coverage of our analysis, we first built a comprehensive catalogue of CRs detected across the entire prokaryotic phylogeny (Fig. 1). Species genomes were retrieved from the proGenomes v2 databases (31). Unlike the NCBI Taxonomy database, which is not an authoritative source for nomenclature or classification (32), proGenomes2 data do not rely on taxonomic names to identify species. Instead, each species-representative genome in proGenomes is delineated based on the evolutionary distances calculated between universally conserved genes present in nearly all organisms (32, 33). To establish links between CRs and the plant-associated lifestyle, we compiled three manually curated databases of PAB (see Materials and Methods): (i) PAB-broad, a reference database of 960 organisms found in multiple plant environments including leaves, roots, and rhizospheric soil; (ii) PAB-phyto, a subset database of 119 species including only known phytopathogens; and (iii) PAB-symb, which groups 192 plant symbionts. Using HMM-based searches, we then mined all the sequences containing the MCPsignal domain in the 11,806 species-representative genomes from the proGenomes database, compiling a global catalogue of 82,277 CR sequences from 5,546 genomes (see Data Set S3 in the supplemental material). This confirms the broad distribution of CRs, with 47% of the representative genomes containing at least one chemotactic receptor.

10.1128/mSystems.00951-21.6 DATA SET S3 List of the 5,546 species-representative genomes in the proGenomes2 database having at least one CR. Download Data Set S3, XLSX file, 0.4 MB. Copyright © 2021 Sanchis-López et al. 2021 Sanchis-López et al. https://creativecommons.org/licenses/by/4.0/ This content is distributed under the terms of the Creative Commons Attribution 4.0 International license.

PAB species possessed almost twice as many CRs per genome (22.86) as those species not classified as plant associated (12.94), with the subset of phytopathogens showing the highest number (27.29). No CRs were predicted in 178 out of the 960 PAB genomes, indicating that more than 81% of PABs possess at least one CR gene, a percentage largely superior to the bacterial average (47%). From all species considered in this study, 36 PAB genomes stood out by their high content of CRs (Table 1), most notably the following: (i) 14 genomes from the Pseudomonas genus (49 to 60 CRs), including the well-known plant pathogens P. syringae and P. savastanoi (34), and (ii) 9 genomes from the *Herbaspirillum* genus (52 to 67 CRs), a group of betaproteobacteria that endophytically colonize gramineous species, thereby promoting their growth (35).

**TABLE 1:** List of PAB with the highest number of predicted CRs

TaxId	Biosample	Representative species	No. of CRs	PAB-phyto^*a*^	PAB-symb^*b*^
1078773	SAMN04334956	Herbaspirillum rubrisubalbicans M1	67		
1144319	SAMN00839627	*Herbaspirillum* sp. CF444	67		
964	SAMN03779333	Herbaspirillum seropedicae	65		
193	SAMN02982994	Azospirillum lipoferum	65		
286727	SAMN02982917	Azospirillum oryzae	64		
346179	SAMN03785417	Herbaspirillum rhizosphaerae	62		
864073	SAMN02471292	Herbaspirillum frisingense GSF30	62		
237610	SAMN05860868	Pseudomonas psychrotolerans	60		
288000	SAMN02598359	*Bradyrhizobium* sp. BTAi1	60		S
92645	SAMN06130964	Herbaspirillum frisingense	59		
1175306	SAMN02469572	*Herbaspirillum* sp. GW103	59		
1121033	SAMN02440867	Azospirillum halopraeferens DSM 3675	58		
169679	SAMN05170519	Clostridium saccharobutylicum	58		
29438	SAMN03837775	Pseudomonas savastanoi	57	P	
1262470	SAMN03010392	Herbaspirillum hiltneri N3	55		
582667	SAMN05192568	Methylobacterium pseudosasicola	54		
50340	SAMN05216581	Pseudomonas fuscovaginae	54		
1001585	SAMN02603190	Pseudomonas mendocina NK-01	54		
1749078	SAMN04216969	Pseudomonas sp. EpS/L25	53		
1190415	SAMN05216593	Pseudomonas asturiensis	53		
50340	SAMN03100370	Pseudomonas fuscovaginae	53		
129140	SAMN03976254	Pseudomonas syringae pv. tagetis	52	P	
294	SAMN04992557	Pseudomonas fluorescens	52		
1855289	SAMN05216319	*Duganella* sp. CF402	52		
1144342	SAMN00839653	*Herbaspirillum* sp. YR522	52		
47885	SAMN03365871	Pseudomonas oryzihabitans	51		
205918	SAMN02604347	Pseudomonas syringae pv. syringae B728a	51	P	
1907416	SAMN05880558	*Aeromonas* sp. RU39B	51		
693986	SAMN03075686	Methylobacterium oryzae CBMB20	50		
1736267	SAMN04151647	Pseudomonas sp. Leaf127	50		
114615	SAMEA3138227	*Bradyrhizobium* sp. ORS 278	50		S
1028989	SAMD00019511	Pseudomonas sp. StFLB209	50		
80867	SAMN04009978	Acidovorax avenae	50	P	
1122963	SAMN02440654	Pleomorphomonas oryzae DSM 16300	50		
223283	SAMN02604017	Pseudomonas syringae pv. tomato DC3000	49	P	
1245469	SAMD00061052	Bradyrhizobium oligotrophicum S58	49		S

a P, phytopathogen.

b S, plant symbiont.

### Classifying chemoreceptors according to their ligand binding domain.

**FIG 2:**
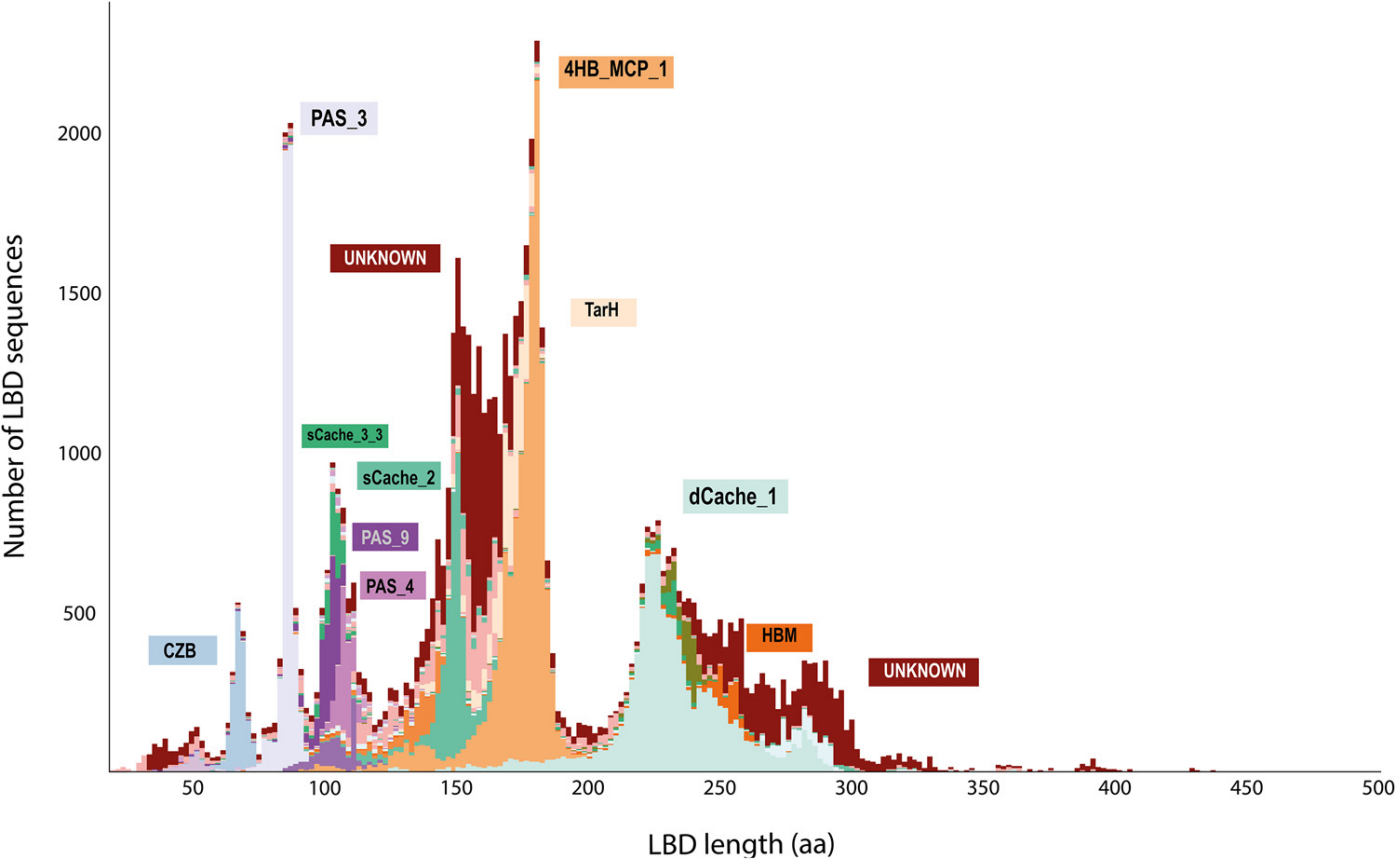
Length distribution of the LBDs. The analysis was conducted on 72,480 LBDs, and the predominant LBD types within each of the main peaks are indicated. Only LBDs shorter than 500 amino acids (aa) are represented.

As the ecological relevance of CRs is mostly defined by their LBD region, we explored whether sequence segments corresponding to the LBD, rather than the full-length CR sequences, were related to a plant-associated lifestyle. To maximize the number of LBD sequences included in our analysis and not limit this to known LBD types from the Pfam database (7), we inferred LBDs based on the domain architecture of each CR. First, we extracted LBD sequences from the whole set of 82,277 CRs. Next, and given the high variability in the domains that could be considered LBDs, we identified putative LBDs using three different strategies: (i) detecting sequence regions matching any known domain other than the MCPsignal or HAMP, (ii) locating sequence regions flanked by two TM regions, and (iii) taking domains between the N-terminus and a single TM region. In total, we retrieved 72,480 putative LBD sequences, which could be fitted into three main groups based on their length (Fig. 2). The first group includes LBDs with a size between 60 and 110 amino acids, containing 21% of all the LBDs detected. The most abundant LBD family within this size range was PAS_3. The second group, comprising LBDs from 130 to 200 amino acids, contained over 45% of all LBDs and included 4HB_MCP_1 as the predominant family. The third group, comprising LBD lengths between 220 and 299 amino acids, covers 26% of all LBDs and has dCache_1 as the most abundant LBD family. Only 8% of all the LBDs detected fell outside these three size ranges, and the three most abundant LBDs were 4HB_MCP_1 (17.6%), dCache_1 (15.5%), and PAS_3 (9.2%).

**FIG 3:**
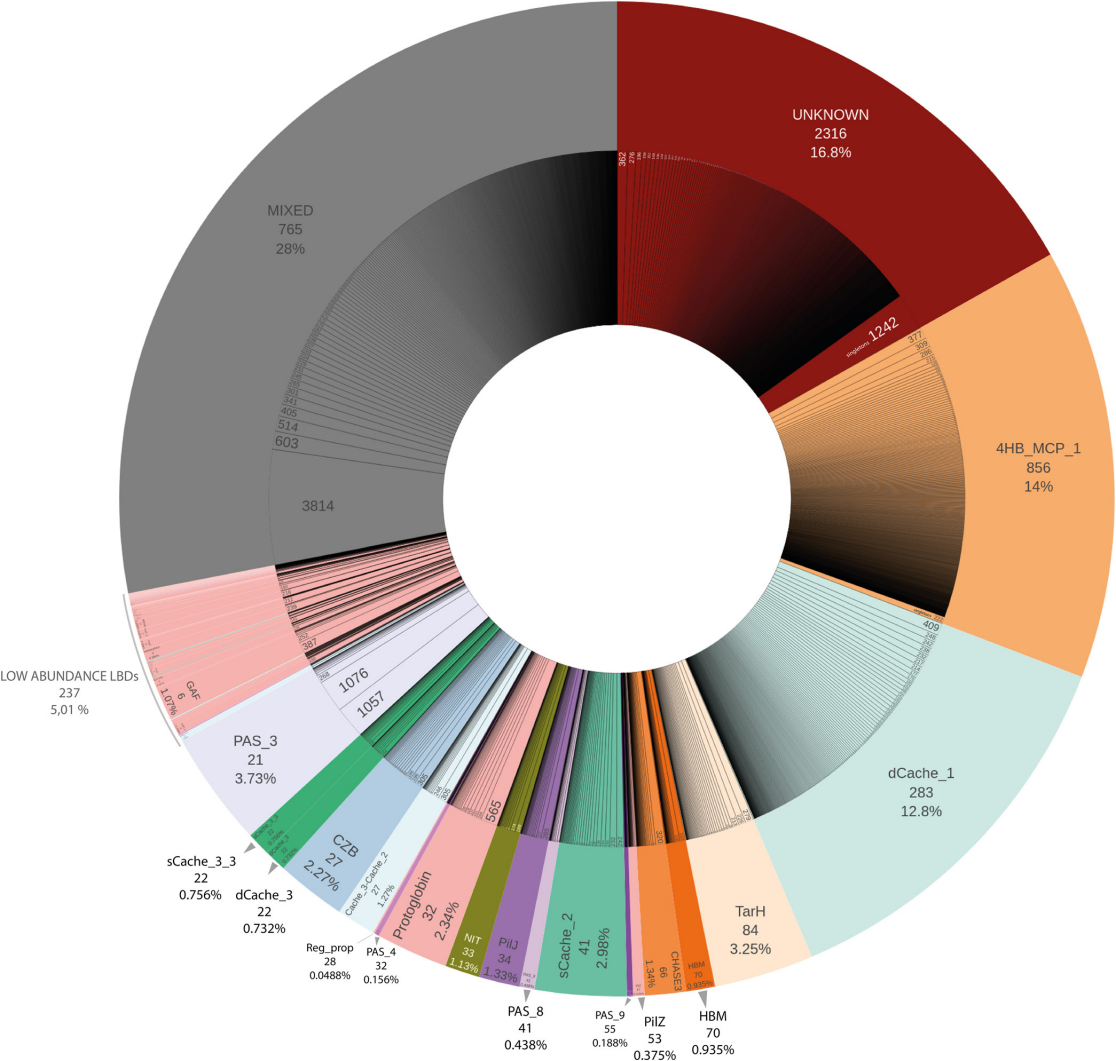
Visual representation of the abundance of the LBD families. The outer donut of the chart represents the distribution of each LBD type and its relative abundance (in percentage of sequences), and the number of clusters with at least 90% of their sequences sharing the same LBD type, as defined by the Pfam signature. The LBDs are sorted according to the number of clusters within each LBD type. The inner donut of the chart represents all the clusters included within each LBD category, indicating the number of sequences contained in each subfamily. All singletons are merged in the last section of each LBD type (e.g., LBDs classified as “Unknown” have 1,242 singletons, that is, clusters containing only one sequence). “Mixed clusters” are those that do not reach the 90% threshold of sequences with the same Pfam model per cluster. “Low-abundance LBDs” include those LBD types that group into fewer than 12 “compact clusters.”

We next investigated whether LBDs could be classified into broader sequence homology clusters, each representing a group of LBD sequences sharing a common evolutionary origin. Using relaxed homology thresholds (E value ≤10^−3^, 50% coverage, 20% amino acid identity), we grouped all 72,480 LBD sequences into 5,149 family clusters (Data Set S4), of which 3,307 contain more than 1 sequence. This *de novo* clustering approach might not be adequate for a detailed functional characterization of LBDs, as single residue changes have been shown to modify LBD ligand affinities (36–38). Nevertheless, each of our LBD clusters could be interpreted as an independent LBD type, with implicit levels of functional and ecological conservation. In fact, our approach consistently recovered all known LBD types and distributed them into 2,068 compact clusters where 90% of their members belonged to the same Pfam domain family (Table S1). Moreover, our clustering strategy allowed us to split large LBD families into finely grained subcategories (Fig. 3). For example, despite 4HB_MCP and Cache-like being present at similar levels in the initial CR sequence database, the number of derived clusters differs significantly, namely, 20.9% for 4HB_MCP compared to 8.3% for Cache-like. In the case of 4HB_MCP_1, the 10,034 sequences group into 856 different clusters compared to the 283 clusters for the 9,162 dCache_1 sequences, indicating higher sequence conservation in the latter. The situation is even more drastic in the case of PAS_3 LBDs, where 2,675 sequences group into just 21 clusters (Table S1), indicating a very low degree of diversity.

10.1128/mSystems.00951-21.2 TABLE S1 Distribution of LBD types among “compact” LBD clusters (at least 90% of their sequences sharing the same LBD type). Download Table S1, PDF file, 0.06 MB. Copyright © 2021 Sanchis-López et al. 2021 Sanchis-López et al. https://creativecommons.org/licenses/by/4.0/ This content is distributed under the terms of the Creative Commons Attribution 4.0 International license.

10.1128/mSystems.00951-21.7 DATA SET S4 List of all CRs identified in 5,546 species-representative genomes obtained from the proGenomes2 database indicating their cluster classification and the amino acid coordinates for their predicted domains. Download Data Set S4, XLSX file, 6.9 MB. Copyright © 2021 Sanchis-López et al. 2021 Sanchis-López et al. https://creativecommons.org/licenses/by/4.0/ This content is distributed under the terms of the Creative Commons Attribution 4.0 International license.

Notably, an important fraction (45%) of the LBD clusters inferred could not be confidently associated with any previous family of Pfam domains, since more than 90% of their LBD sequences did not match to any known domain signature, suggesting the existence of a large number of unknown LBD types.

### Identifying PAB-specific ligand binding domains.

To identify LBD families specific to a plant-associated lifestyle, we analyzed each LBD cluster and calculated the corresponding percentage of PAB species therein, which we referred to as the degree of plant specificity (DPS; see Materials and Methods). For each LBD cluster, we calculated three DPS values, based on three databases of PAB species: (i) DPS-broad, calculated based on the PAB-broad reference database; (ii) DPS-phyto, based on the PAB-phyto subset; and (iii) DPS-symb, using the PAB-symb subgroup as a reference. In all cases, the DPS values ranged from 0% (no LBD family observed in the corresponding PAB database) to 100% (the LBD cluster includes only species from a given reference PAB database). From the 3,307 LBD nonsingleton clusters, we identified 419 and 139 clusters with a DPS-broad score of ≥50%, and ≥80%, respectively. Similarly, many LBD clusters showed high specificity in the stricter PAB reference databases (Data Set S5).

10.1128/mSystems.00951-21.8 DATA SET S5 List of the 5,149 clusters of LBDs indicating their DPS-broad, DPS-phyto, and DPS-symb. Download Data Set S5, XLSX file, 0.3 MB. Copyright © 2021 Sanchis-López et al. 2021 Sanchis-López et al. https://creativecommons.org/licenses/by/4.0/ This content is distributed under the terms of the Creative Commons Attribution 4.0 International license.

To further validate our findings, we cross-linked our predictions with experimental data from previous studies (39–43). In particular, we found that CRs with increased expression *in planta*, and particularly those required for full bacterial virulence, belonged to high-DPS clusters (Table 2). This list includes CRs that are upregulated in Dickeya dadantii 3937 and Pectobacterium carotovorum WPP14, two soft-rot bacterial strains (39); Dickeya dianthicola RNS04.9, which grows on macerated potato tubers (40); and Xanthomonas fragariae, which grows on strawberry leaves (41). Similarly, we found several CRs with very high DPS values (80%) that were shown to be relevant in Xanthomonas citri virulence (42) or required for fitness of Pseudomonas savastanoi pv. savastanoi in olive knots (43). Taken together, these data support the validity of our approach to identify CRs that are relevant for a plant-associated lifestyle.

**TABLE 2:** CRs predicted to be involved in plant-bacterium interactions

CR gene ID (in the original source)	LBD type	DPS (%)	DPS-phyto (%)	DPS-symb (%)	Cluster no.	Amino acid identity (%) to representative LBDs from the database	Bacterial species and strain	Reference
ABF-0014824	TarH	100	100	0	932	100	Dickeya dadantii 3937	39
ABF-0015168	TarH	68.09	40.43	0	179	100	Dickeya dadantii 3937	39
ABF-0016115	HBM	63.64	48.48	0	409	100	Dickeya dadantii 3937	39
ABF-0016585	TarH	51.09	14.60	0.73	42	100	Dickeya dadantii 3937	39
ABF-0017097	Unknown	80.00	80.00	0	838	100	Dickeya dadantii 3937	39
ABF-0017674	4HB_MCP_1	100	87.50	0	630	100	Dickeya dadantii 3937	39
ABF-0019851	TarH	61.22	42.86	0	123	100	Dickeya dadantii 3937	39
ABF-0019855	TarH	61.22	42.86	0	123	100	Dickeya dadantii 3937	39
ABF-0020431	sCache_2	34.55	12.73	0	233	100	Dickeya dadantii 3937	39
DDI_0843	dCache_1	38.61	10.13	1.27	51	100	Dickeya dianthicola RNS04.9	40
DDI_0932	sCache_2	34.55	12.73	0	233	100	Dickeya dianthicola RNS04.9	40
DDI_1647	TarH	61.22	42.86	0	123	88.97	Dickeya dianthicola RNS04.9	40
DDI_1649	TarH	61.22	42.86	0	123	100	Dickeya dianthicola RNS04.9	40
DDI_2258	HBM	100	100	0	792	100	Dickeya dianthicola RNS04.9	40
DDI_4092	4HB_MCP_1	100	87.50	0	630	100	Dickeya dianthicola RNS04.9	40
ADT-0000027	HBM	63.64	48.48	0	409	96.03	Pectobacterium carotovorum WPP14	39
ADT-0000661	sCache_2	38.46	12.82	0	160	99.30	Pectobacterium carotovorum WPP14	39
ADT-0001320	TarH	51.09	14.60	0.73	42	98.84	Pectobacterium carotovorum WPP14	39
ADT-0001602	TarH	56.99	29.03	0	116	94.15	Pectobacterium carotovorum WPP14	39
ADT-0001887	TarH	61.22	42.86	0	123	97.59	Pectobacterium carotovorum WPP14	39
ADT-0002104	TarH	100	100	0	932	100	Pectobacterium carotovorum WPP14	39
ADT-0003152	TarH	68.09	40.43	0	179	91.15	Pectobacterium carotovorum WPP14	39
ADT-0003245	4HB_MCP_1	100	87.50	0	630	97.40	Pectobacterium carotovorum WPP14	39
ADT-0003418	Unknown	80.00	80.00	0	838	95.60	Pectobacterium carotovorum WPP14	39
PSA3335_17610	Unknown	87.50	50.00	0	835	100	Pseudomonas savastanoi NCPPB3335	43
XAC1892	Unknown	100	100	0	846	86.77	Xanthomonas citri subsp. *citri* XHG3	42
XAC2448	4HB_MCP_1	39.23	14.62	0	77	98.88	Xanthomonas citri subsp. *citri* XHG3	42
NBC2815_01024	4HB_MCP_1	92.00	84.00	0	549	100	Xanthomonas fragariae IPO 3485	41
NBC2815_02005	4HB_MCP_1	60.53	42.11	0	353	100	Xanthomonas fragariae IPO 3485	41
NBC2815_02008	4HB_MCP_1	88.46	88.46	0	273	100	Xanthomonas fragariae IPO 3485	41
NBC2815_02009	4HB_MCP_1	82.14	75.00	0	340	100	Xanthomonas fragariae IPO 3485	41

Interestingly, we also found that many PAB-specific clusters (41.75%) are formed by proteins of unknown LBD type, suggesting the presence of a significant number of uncharacterized LBD types. Excluding unknown LBD families, the most common domains among high-DPS clusters are 4HB_MCP_1 (26%), TarH (4.5%), and HBM (4%) (Table 3). It is remarkable that the three domain families form four-helix bundle structures (37, 38). The case of the HBM and TarH domains is particularly interesting, as the majority of sequences that belonged to these categories concentrated in very few high-DPS clusters: 57.0% (516/906) of all HBM sequences are grouped into 23 high-DPS clusters, and 36.7% (1,042/2,840) of all TarH sequences are grouped into 26 high-DPS clusters. This indicates a strong association of the TarH and HBM domains with the plant-associated lifestyle. In contrast, despite being the second most abundant LBD in bacteria (Table S2), the dCache_1 domain was not very abundant in PAB.

**TABLE 3:** Distribution of LBD types among clusters with high DPS (≥50%)

LBD type	No. of clusters	% of clusters over total^*a*^	No. of LBD sequences with the indicated domain^*b*^	Avg no. of LBD sequences per cluster
Unknown	243	41.75	2,053	8.45
4HB_MCP_1	151	25.95	2,642	17.50
TarH	26	4.47	1,042	40.08
HBM	23	3.95	516	22.43
CHASE3	9	1.55	142	15.78
PilZ	7	1.20	39	5.57
PAS_9	6	1.03	7	1.17
sCache_2	4	0.69	245	61.25
NIT	4	0.69	79	19.75
Protoglobin	3	0.52	153	51
PAS_8	3	0.52	9	3
PAS_3	3	0.52	82	27.33
dCache_1	3	0.52	151	50.33
Cache_3-Cache_2	3	0.52	79	26.33
PAS_4	2	0.34	4	2
CHASE4	2	0.34	3	1.50
Usher	1	0.17	1	1
Tox-URI2	1	0.17	1	1
SURF1	1	0.17	1	1
SOR_SNZ	1	0.17	1	1
sCache_3_3	1	0.17	2	2
Porin_4	1	0.17	1	1
Peripla_BP_5	1	0.17	1	1
PAS_7	1	0.17	19	19
PapC_N	1	0.17	1	1
Glyco_hydro_2_N	1	0.17	1	1
Glyco_hydro_106	1	0.17	1	1
FHIPEP	1	0.17	1	1
DUF4077	1	0.17	5	5
dCache_3	1	0.17	71	71
CBS	1	0.17	2	2
Asparaginase	1	0.17	1	1
ABC_tran	1	0.17	1	1
5TM-5TMR_LYT	1	0.17	20	20
Total	510		7,377	

a Percentages are calculated over the total number of LBD clusters with at least 90% of their sequences sharing the same LBD type. These total clusters comprise more than 88% of the total number of clusters in this work.

b Sum of the total number of sequences sharing the same domain type found in the indicated clusters.

10.1128/mSystems.00951-21.3 TABLE S2 Distribution of LBD types and domain models in the total LBD data set. Download Table S2, PDF file, 0.04 MB. Copyright © 2021 Sanchis-López et al. 2021 Sanchis-López et al. https://creativecommons.org/licenses/by/4.0/ This content is distributed under the terms of the Creative Commons Attribution 4.0 International license.

### Phylogenetic versus ecological signal in PAB-specific ligand binding domains.

**FIG 4:**
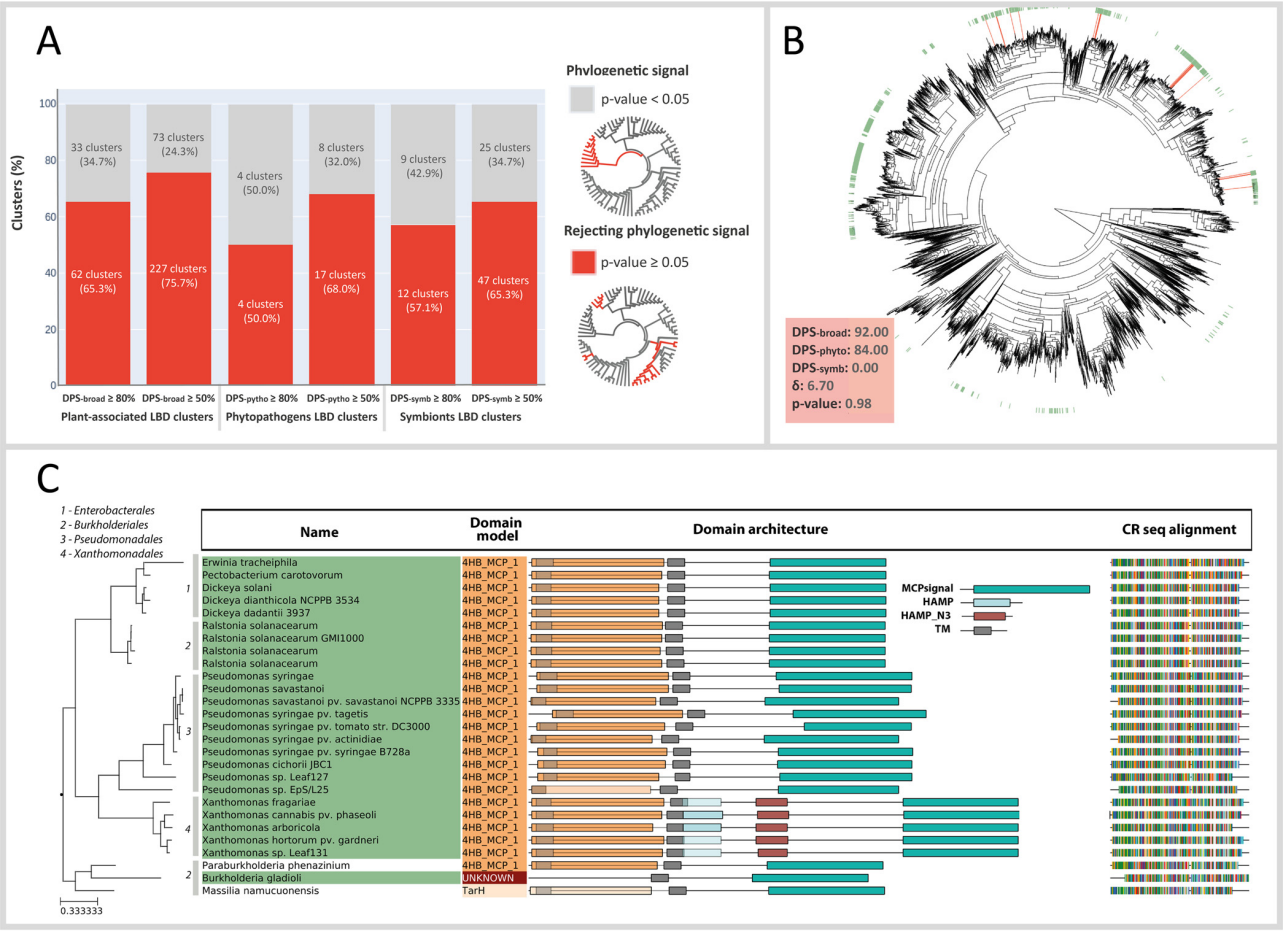
Phylogenetic signal detection in plant-associated LBD clusters. (A) Proportion of the significant phylogenetic signal among LBD clusters enriched in PAB-broad, PAB-phyto, and PAB-symb species within two thresholds (≥50%, ≥80%). The significance through a *P* value test with 100 iterations, *P* value ≥0.05 rejects the null hypothesis of a phylogenetic signal (see Materials and Methods). (B) LBD cluster 549 (in red) distributed according to the chemotactic species phylogeny (5,763 representative species). The green leaves of the tree represent the PAB species. DPS, δ, and *P* values for this LBD cluster are represented in the lower left box. (C) Phylogeny representation of LBD cluster 549, containing 27 LBD sequences distributed across 4 orders (numbered 1 to 4 in the tree). The domain architecture prediction is shown for each of the CRs.

Intrigued by the potential ecological significance of PAB-specific LBD clusters, we further tested whether their taxonomic distribution is due to the phylogenetic signal of the underlying species, or if it might be driven by additional ecological factors. To address this issue, we reconstructed the complete phylogeny of the 11,806 species considered here (see Materials and Methods) and used it to assess the taxonomic distribution of each individual LBD cluster. Using the δ-approach (44), we found that the majority (75.7%) of plant-associated LBD types (DPS ≥50%) did not follow the expected phylogenetic signal. In contrast, the taxonomic distribution of most PAB-specific LBDs was scattered over the global bacterial phylogeny (Fig. 4). This observation was consistent for the three PAB reference databases considered in this study, using stricter DPS cutoffs, and even when the species lacking CR genes were excluded from the analysis (Fig. S1).

10.1128/mSystems.00951-21.1 FIG S1 Phylogenetic signal detection in plant-associated LBD clusters using the phylogeny of chemotactic species tree. Proportion of significant phylogenetic signal among LBD clusters enriched in PAB-broad, PAB-phyto, and PAB-symb species within two thresholds (≥50%, ≥80%). The reference phylogeny used in this analysis excluded any species without CRs in their genome. Download FIG S1, TIF file, 0.5 MB. Copyright © 2021 Sanchis-López et al. 2021 Sanchis-López et al. https://creativecommons.org/licenses/by/4.0/ This content is distributed under the terms of the Creative Commons Attribution 4.0 International license.

Overall, the lack of phylogenetic signal for most of the LBD clusters, together with the fact that the LBDs tested are enriched in PAB species, suggests that the evolution of the sensory machinery of bacterial species might be at least partially driven by ecological pressures. This should allow the use of particular LBD clusters, even if functionally undefined, as lifestyle biomarkers. This issue is best illustrated by the LBD cluster 549 (Fig. 4B and C), which contains 27 CRs from broadly distributed bacterial families and orders, while retaining a high plant-association signal (DPS-broad >80%).

### LBD profiles per genome.

**FIG 5:**
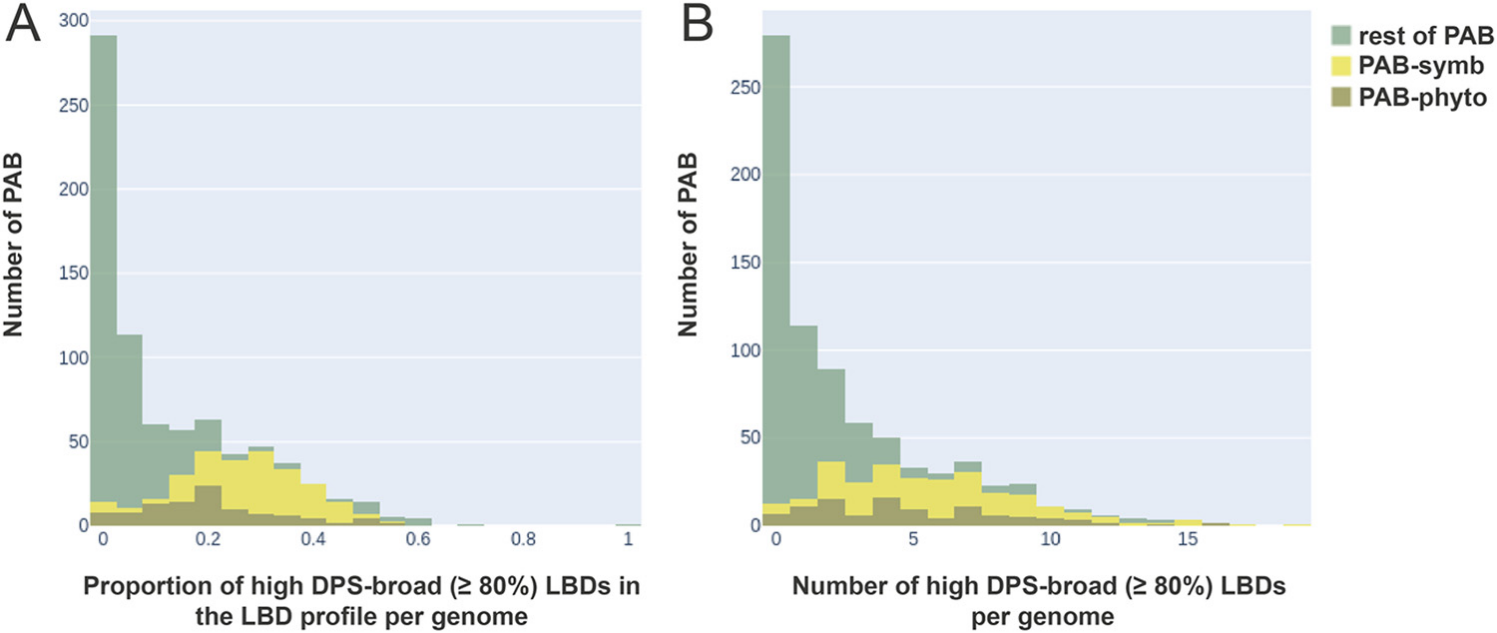
Distribution of the high-plant-specificity LBDs in the PAB species profiles per genome. (A) Calculation of the proportion of high-DPS-broad (≥80%) LBDs in the total number of LBDs present in each species. The graph illustrates the distribution of the number of species according to the proportional ranges, plotting the species count as PAB-symb (yellow), PAB-phyto (brown), and the rest of PAB (green). (B) Absolute number of LBDs with a DPS-broad value of ≥80% in each PAB genome. The graph illustrates the distribution of the number of species as an absolute count of high-DPS LBD ranges. The species count is plotted as PAB-symb (yellow), PAB-phyto (brown), and the rest of PAB (green).

To investigate whether the profile of LBD clusters per genome could be informative about the plant-associated bacterial lifestyle, we studied the full repertoire of CRs among different PAB species. The genomes from the PAB species not only contained more CRs than those of non-PAB species, but also, many of their CRs could be considered highly specific to plant-related environments. In fact, assessing the LBD profiles per genome showed that microorganisms with a pronounced plant-associated lifestyle (i.e., PAB-symb and PAB-phyto) harbor more specific CRs than other PAB species (Fig. 5). On average, 28% and 20% of plant-symbiont and plant-phytopathogen CRs, respectively, are highly specific (DPS-broad >80%). In contrast, other PAB with a less pronounced plant-associated lifestyle, like nonsymbiont and nonphytopathogen plant-associated species, contained significantly fewer specific CRs (6%) (Fig. 5). Taken together, this information reinforces the idea that the repertoire of CRs has been partially shaped by niche adaptation, with more specialized adaptations leading to more specific CRs.

## DISCUSSION

In the present study, we carried out a comprehensive phylogenomic analysis of the full repertoire of CRs from a wide collection of microbial genomes, classifying them according to their LBDs. To maximize the representativeness of our study, we used more than 82,000 species-level CR sequences from 11,000 species-representative genomes, significantly expanding the scope of previous works (7, 15, 45), in terms of both the number of sequences examined and the phylogenetic coverage. To achieve this, we developed a novel method to extract LBDs and classified them based on a *de novo* homology-based clustering approach, departing from the traditional classification of CRs centered around their general protein topology (15, 45–47) or on known LBD domain searches (7). This approach allowed us to identify many new potential LBD types, suggesting that the chemosensing landscape remains largely unexplored. Additionally, we believe that our strategy delineating large LBD families into finely grained subcategories could provide further information (Fig. 3). Moreover, by classifying CRs based on their putative LBD type, for the first time we were able to quantify to what extent the chemosensory activity of PAB is linked to lifestyle.

Considering the enormous variety of LBDs at sensor proteins, establishing the forces that have driven their evolution is an important question that was never specifically addressed. To our knowledge, we present here the first clear demonstration showing that environmental factors play an important role in the selection and evolution of LBDs. We found that the specificity of LBDs to a plant-associated lifestyle could not be explained by just a phylogenetic signal, since the taxonomic distribution of most PAB-specific LBD types was scattered over the microbial phylogeny, which at times covered different orders and phyla. This indicates that the selection of the certain CRs might indeed be guided by ecological factors, opening the possibility of identifying lifestyle biomarkers.

We also found that bacterial species more tightly associated with plant environments (such as plant symbionts and phytopathogens) tend to have stronger lifestyle specificity signals in their CR repertory. For instance, plant symbionts had the largest number of PAB-specific LBDs per genome, followed by phytopathogens, with both showing significantly higher ratios than generic soil microbiota. It appears likely that even stronger links between the chemosensory capabilities of bacteria and their lifestyle will be detected in the future as more data become available on new organisms (e.g., via metagenomics sequencing) and on their niche adaptation (i.e., plant-tissue specificity).

These findings thus offer a number of research opportunities in the field of signal transduction. First, it can be explored whether similar relationships can be observed in CRs of bacteria with a different lifestyle, such as for example those that inhabit or infect the human intestine. Another interesting issue that needs to be addressed is the question whether similar LBD types are shared by members of different sensor protein families. Major families of these receptors are sensor histidine kinases; chemoreceptors; adenylate, diadenylate, and diguanylate cyclases; and certain cAMP, c-di-AMP, and c-di-GMP phosphodiesterases, as well as Ser/Thr/Tyr protein kinases and phosphoprotein phosphatases (48). As the different sensor proteins of a given strain are exposed to the same signals, it appears plausible that the same LBD types might be present in members of different sensor protein families. Several examples have been reported in this direction, such as the specific sensing of nitrate by PilJ-type LBDs of the NarQ-type sensor kinases (49), the McpN chemoreceptor (50), and the PAS domain, universally found in different signal transduction systems (48). It would be of interest to estimate the global occurrence of such cases.

Overall, we believe that our study provides a comprehensive resource for future studies on bacterial chemoreception and that it sets the basis for the identification of novel CRs relevant for bacterium-plant interactions.

## MATERIALS AND METHODS

### Chemoreceptor (CR) sequence retrieval.

From the genomes of 11,806 representative species in the proGenomes2 database (31), 82,277 CR sequences were obtained. The representative species in proGenomes2 are the result of a phylogeny-based classification of all RefSeq (51) genomes, where species delineation is based on a systematic phylogenetic threshold (i.e., <95% divergence in 40 universal marker genes) rather than relying on the NCBI taxonomic names. Although this might lead to inconsistencies with the current NCBI Taxonomy names for strains and species, it better represents the genomic definition of species, as well as providing a standardized classification system (33, 52). To identify CRs in our set of representative genomes, all the sequences matching the MCPsignal Pfam domain signature (PF00015) were retrieved using HMMER 3.1b2 (53), Pfam-A 31.0 (54), and the specific gathering threshold provided for the MCPsignal HMM Pfam model. Multiple hits were resolved by retaining the match with the highest bit score. In analogy to previous studies (7, 55, 56), the presence of an MCPsignal domain in the sequence was the only criterion used for CR identification.

### Ligand binding domain (LBD) extraction.

For each CR sequence, transmembrane regions (TMs) were predicted using TMHMM2 (57). The position of the TM region(s) was used to infer the putative extracellular LBD regions, which were subsequently annotated using the Pfam domain database. When no significant Pfam matches were found, LBD sequences were labeled as “unknown.” Two different topologies of extracellular LBDs were considered: (i) sequence regions flanked by two TM regions and (ii) sequence regions located between one TM and the N-terminal sequence. In both cases, sequences shorter than 30 amino acids were discarded. Intracellular LBD regions, as well as potentially overlooked extracellular LBDs (e.g., due to undetected TMs), were inferred based on the detection of Pfam domains other than the MCPsignal and HAMP domains. Pfam mappings were performed using HMMER (53) searches as implemented in eggNOG-mapper v.2.0.5 (53, 58). When more than two domains mapped to the same region, the best hit was selected. The final data set contained 72,480 LBD sequences.

### Clustering of LBD sequences.

*De novo* homology-based clustering of the 72,480 LBD sequences was inferred using MMseqs2 (59) with an E value threshold of 0.01, 20% minimum identity, and 50% minimum query coverage. These parameters were chosen to maximize remote homology detection and to infer LBD clusters with broad phylogenetic divergence (i.e., distant homologues) while still grouping sequences with a common evolutionary origin. The MMseqs2 command used was “mmseqs cluster -c 0.2 –min-seq-id 0.5 –cov-mode 2”.

### Construction of the databases for plant-associated bacteria (PAB).

A curated list of PAB was manually curated from the 11,806 representative species. As a first filter, we used the habitat information (i.e., “host plant-associated” label) provided by proGenomes2, which is based on the PATRIC database (31). The resulting list was reviewed manually to exclude uncertain or incorrectly annotated entries by checking their metadata and associated literature. Additionally, we included other known plant-associated species on the list that were missed by the PATRIC database but that were considered PAB based on published data. In total, we identified 960 reference species (PAB-broad) that could be considered related to the plant environment. From this list, we extracted two subdatabases (see Data Set S1 in the supplemental material): phytopathogens (PAB-phyto, 119 members) and plant symbionts (PAB-symb, 192 members).

10.1128/mSystems.00951-21.4 DATA SET S1 List of the bacterial TaxIds included in the plant-associated bacteria (PAB) database. Download Data Set S1, XLSX file, 0.1 MB. Copyright © 2021 Sanchis-López et al. 2021 Sanchis-López et al. https://creativecommons.org/licenses/by/4.0/ This content is distributed under the terms of the Creative Commons Attribution 4.0 International license.

### Degree of plant specificity (DPS).

A specificity score for plant association was calculated for each LBD type based on the proportion of PAB species present in each LBD cluster. We calculated three score values, which we refer to as the degree of plant specificity (DPS), depending on the PAB reference database used: DPS-broad, the proportion of PAB-broad species in each LBD cluster; DPS-phyto, the proportion of PAB-phyto species; and DPS-symb, the proportion of PAB-symb species.

### Phylogenetic tree reconstruction and visualization.

Multiple sequence alignments were built for each cluster using Clustal Omega v1.2.4 (60), and phylogeny was inferred by IQ-Tree v1.6.12 using the default parameters (61). The trees were further analyzed and visualized using ETE3 v3.0 (62), with custom Python scripts integrating the annotations of each sequence for its taxonomy, domain architecture, sequence alignment, and plant-specificity prediction (DPS).

### Phylogenetic signal tests.

The phylogenetic signal tests were performed using the δ-approach (44), a phylogenetic analogue of the Shannon entropy that measures the degree of phylogenetic signal between a categorical trait (trait vector) and a phylogeny (metric-tree). We used the δ-approach to specifically test the null hypothesis that a given taxonomic distribution of an LBD follows the phylogenetic signal of the underlying species, which provided us with a *P* value for each LBD cluster. We applied 100 iterations per test and set the *P* value threshold at 0.05.

The species phylogeny used as a reference in all the tests was reconstructed using the ETE3 (62) supermatrix-based workflow and a concatenated alignment of 40 universal marker genes (63) extracted from the 11,806 species-representative genomes using the FetchMG tool (64). Multiple sequence alignments were inferred using Clustal Omega v1.2.4 (60), and phylogenetic reconstruction was performed with FastTree v2.1 (65). Moreover, an alternative species phylogeny including only genomes with at least one CR was reconstructed using the same methodology. As the δ-statistic has poor sensitivity in detecting the phylogenetic signal for small taxon sample sizes (<20 taxa), LBD clusters mapping to reference phylogenetic tree nodes smaller than 20 leaves were discarded from the analysis (Data Set S2).

10.1128/mSystems.00951-21.5 DATA SET S2 Delta-statistic values to calculate the phylogenetic signal of the LBD clusters. Download Data Set S2, XLSX file, 0.08 MB. Copyright © 2021 Sanchis-López et al. 2021 Sanchis-López et al. https://creativecommons.org/licenses/by/4.0/ This content is distributed under the terms of the Creative Commons Attribution 4.0 International license.
