# Supplementary material for: Prevalence and Specificity of Chemoreceptor Profiles in Plant-Associated Bacteria
Source: mSystems. 2021 Sep 21;6(5):e00951-21. doi: 10.1128/mSystems.00951-21 (PMC8547431; doi:10.1128/mSystems.00951-21)
Supplement: TABLE S1 [file msystems.00951-21-st001.pdf]

Table S1. Distribution of LBD types among 'compact' LBD clusters (at least 90% of their sequences sharing the same LBD type).

LBD type	Number of compact clusters	% of clusters over total [a]	Number of LBD sequences with the indicated domain [b]	Average number of LBD sequences per cluster [c]
UNKNOWN clusters	2316	44.98	12072	5.2
4HB_MCP_1	856	16.62	10034	11.7
dCache_1	283	5.50	9162	32.4
TarH	84	1.63	2328	27.7
HBM	70	1.36	671	9.6
CHASE3	66	1.28	959	14.5
PilZ	57	1.11	270	4.7
PAS_9	55	1.07	135	2.5
PAS_8	41	0.80	350	8.5
sCache_2	41	0.80	2134	52.0
PilJ	34	0.66	1072	31.5
NIT	33	0.64	810	24.5
PAS_4	32	0.62	112	3.5
Protoglobin	32	0.62	1682	52.6
Reg_prop	28	0.54	38	1.4
Cache_3-Cache_2	27	0.52	914	33.9
CZB	27	0.52	1629	60.3
dCache_3	22	0.43	525	23.9
sCache_3_3	22	0.43	542	24.6
PAS_3	21	0.41	2675	127.4
sCache_3_2	12	0.23	142	11.8
PAS	10	0.19	29	2.9
7TMR-DISM_7TM	8	0.16	43	5.4
CBS	8	0.16	65	8.1
CHASE4	8	0.16	23	2.9
GAF_2	8	0.16	214	26.8
Peripla_BP_4	8	0.16	50	6.3
GAF	6	0.12	766	127.7
MHYT	6	0.12	36	6
dCache_2	5	0.10	11	2.2
DUF3365	5	0.10	206	41.2
SBP_bac_3	5	0.10	60	12
CHASE8	4	0.08	22	5.5
cNMP_binding	4	0.08	7	1.8
Diacid_rec	4	0.08	90	22.5
Phosphonate-bd	4	0.08	74	18.5
PocR	4	0.08	166	41.5
DUF2222	3	0.06	3	1
Fer4	3	0.06	25	8.3
FIST	3	0.06	219	73
Hemerythrin	3	0.06	276	92
Rhodanese	3	0.06	10	3.3
5TM-5TMR_LYT	2	0.04	26	13
7TMR-DISMED2	2	0.04	10	5
Ammonium_transp	2	0.04	64	32
Fe_hyd_lg_C	2	0.04	124	62
FIST_C	2	0.04	220	110
GGDEF	2	0.04	2	1
HNOB	2	0.04	235	117.5
NMT1_2	2	0.04	4	2
OpuAC	2	0.04	13	6.5
PAS_10	2	0.04	3	1.5
PrpR_N	2	0.04	18	9
Response_reg	2	0.04	7	3.5
SBP_bac_5	2	0.04	64	32
T7SS_ESX_EspC	2	0.04	2	1
5_3_exonuc	1	0.02	1	1
5_3_exonuc_N	1	0.02	1	1
ABC_sub_bind	1	0.02	1	1
ABC_tran	1	0.02	1	1
AhpC-TSA	1	0.02	5	5
AI-2E_transport	1	0.02	1	1
Alanine_zipper	1	0.02	1	1
Asparaginase	1	0.02	1	1
B12-binding	1	0.02	2	2
Bac_globin	1	0.02	6	6
Bac_rhodopsin	1	0.02	1	1
Band_7	1	0.02	1	1
BLUF	1	0.02	1	1
bZIP_2	1	0.02	1	1
Cas_DxTHG	1	0.02	1	1
CBM_4_9	1	0.02	2	2
CHASE	1	0.02	1	1
DctP	1	0.02	8	8
DNA_pol_A_exo1	1	0.02	1	1
DUF1640	1	0.02	1	1
DUF3235	1	0.02	1	1
DUF4077	1	0.02	5	5

DUF4118	1	0.02	1	1
DUF442	1	0.02	1	1
DUF948	1	0.02	2	2
EAL	1	0.02	1	1
EF-hand_11	1	0.02	1	1
EMC3_TMCO1	1	0.02	1	1
Exonuc_VII_L	1	0.02	1	1
Fe-ADH	1	0.02	1	1
Fer4_20	1	0.02	3	3
FeS	1	0.02	78	78
FHIPEP	1	0.02	1	1
FRB_dom	1	0.02	1	1
GFO_IDH_MocA	1	0.02	6	6
Globin	1	0.02	20	20
Glyco_hydro_106	1	0.02	1	1
Glyco_hydro_2_N	1	0.02	1	1
H_kinase_N	1	0.02	2	2
He_PIG	1	0.02	8	8
HHH_5	1	0.02	2	2
HTH_ParB	1	0.02	1	1
Htr2	1	0.02	7	7
IGPD	1	0.02	1	1
Ion_trans_2	1	0.02	1	1
LXG	1	0.02	1	1
MASE3	1	0.02	4	4
MASE4	1	0.02	2	2
MerR_1	1	0.02	1	1
Mut7-C	1	0.02	1	1
NADH_4Fe-4S	1	0.02	3	3
NMT1	1	0.02	2	2
PapC_N	1	0.02	1	1
PAS_6	1	0.02	1	1
PAS_7	1	0.02	19	19
Pentapeptide	1	0.02	1	1
Pentapeptide_4	1	0.02	1	1
Peripla_BP_3	1	0.02	4	4
Peripla_BP_5	1	0.02	1	1
Peripla_BP_6	1	0.02	2	2
PhoU	1	0.02	1	1
Porin_4	1	0.02	1	1
Pro_CA	1	0.02	4	4
PTS_EIIC	1	0.02	2	2
Pyr_redox_2	1	0.02	3	3
SBP_bac_1	1	0.02	1	1
SBP_bac_8	1	0.02	26	26
SDH_alpha	1	0.02	1	1
Sensor_TM1	1	0.02	1	1
SOR_SNZ	1	0.02	1	1
SpoIIAA-like	1	0.02	1	1
SURF1	1	0.02	1	1
THF_DHG_CYH	1	0.02	1	1
THF_DHG_CYH_C	1	0.02	1	1
TnpV	1	0.02	1	1
Tox-URI2	1	0.02	1	1
TPR_19	1	0.02	4	4
Transgly	1	0.02	1	1
Ufd2P_core	1	0.02	1	1
UPF0184	1	0.02	1	1
Usher	1	0.02	1	1
VPS28	1	0.02	1	1
Vps53_N	1	0.02	1	1
Y_Y_Y	1	0.02	10	10
YkyA	1	0.02	1	1
TOTAL	4384		51741	

[a] Percentages are calculated over the total number of LBD clusters (5,149).

[b] Sum of the total number of sequences sharing the same domain type found in the indicated clusters.

[c] 'UNKNOWN clusters' are those with 90% or more of their sequences that did not match to any known domain signature in Pfam.

Distribution of LBD types grouped by domain superfamilies (only the three most abundant superfamilies are shown)

Domain superfamily	Generic LBD type	Domain model	% of clusters over total [a]
**4HB_MCP**	Single 4HB	4HB_MCP_1	16.62
		TarH	1.63
		CHASE3	1.28
	Double 4HB	HBM	1.36
		**Subtotal:**	**20.90**
**Cache-like**	Double Cache	dCache_1	5.50
		Cache_3-Cache_2	0.52
		dCache_3	0.43
		dCache_2	0.10
	Single Cache	sCache_2	0.80
		sCache_3_3	0.43
		sCache_3_2	0.23
		Diacid_rec	0.08
		CHASE4	0.16
		CHASE8	0.08
		**Subtotal:**	**8.31**
**PAS**	PAS	PAS_3	0.41
		PAS_9	1.07
		PAS_4	0.62
		PAS_8	0.80
		PAS	0.19
		PAS_7	0.02
		PAS_10	0.04
		PAS_6	0.02
		**Subtotal:**	**3.17**
