# Supplementary material for: Prevalence and Specificity of Chemoreceptor Profiles in Plant-Associated Bacteria
Source: mSystems. 2021 Sep 21;6(5):e00951-21. doi: 10.1128/mSystems.00951-21 (PMC8547431; doi:10.1128/mSystems.00951-21)
Supplement: TABLE S2 [file msystems.00951-21-st002.pdf]

Table S2. Distribution of LBD types and domain models in the total LBD dataset.

Domain superfamily	LBD type	Domain model		No. of LBDs	% of each domain model
4HB_MCP	Single 4HB	4HB_MCP_1		12754	17.597
		TarH		2840	3.918
		CHASE3		1133	1.563
	Double 4HB	HBM		906	1.250
			Subtotal:	*17633*	*24.328*
Cache-like	Double Cache	dCache_1		11216	15.475
		Cache_3-Cache_2		1381	1.905
		dCache_3		598	0.825
		dCache_2		560	0.773
		YkuI_C		5	0.007
	Single Cache	sCache_2		4049	5.586
		sCache_3_3		779	1.075
		sCache_3_2		179	0.247
		Diacid_rec		90	0.124
		CHASE4		35	0.048
		CHASE8		23	0.032
			Subtotal:	*18915*	*26.097*
Unknown	Unknown	UNKNOWN		15776	21.766
PAS	PAS	PAS_3		6704	9.249
		PAS_9		2096	2.892
		PAS_4		1459	2.013
		PAS_8		430	0.593
		PAS		316	0.436
		PAS_7		19	0.026
		PAS_10		3	0.004
		PAS_6		1	0.001
			Subtotal:	*11028*	*15.215*
GAF	GAF	GAF		786	1.084
		GAF_2		232	0.320
		GAF_3		1	0.001
			Subtotal:	*1019*	*1.406*
Protoglobin	Protoglobin	Protoglobin		1682	2.321
		Globin		20	0.028
		Bac_globin		6	0.008
			Subtotal:	*1708*	*2.357*
PBP	Periplasmic Binding Proteins	Phosphonate-bd		74	0.102
		SBP_bac_5		64	0.088
		SBP_bac_3		60	0.083
		Peripla_BP_4		50	0.069
		SBP_bac_8		36	0.050
		OpuAC		13	0.018
		Peripla_BP_5		9	0.012
		DctP		8	0.011
		SBP_bac_1		5	0.007
		Peripla_BP_3		4	0.006
		NMT1_2		4	0.006
		Peripla_BP_6		3	0.004
		NMT1		2	0.003
			Subtotal:	*332*	*0.458*
HNOX-like	Heme and NO binding	HNOB		235	0.324
4Fe-4S	Iron-sulfur cluster binding	FeS		78	0.108
		Fer4_10		54	0.075
		Fer4		46	0.063
		Fer4_7		14	0.019
		Fer4_9		11	0.015
		Fer4_6		3	0.004
		Fer4_20		3	0.004
			Subtotal:	*209*	*0.288*
GPCR-A	GPCR-like	7TMR-DISM_7TM		43	0.059
NADP-Rossman	NAD binding	GFO_IDH_MocA		15	0.021
		Semialdhyde_dh		4	0.006
			Subtotal:	*19*	*0.026*
Gx-transp	Transporter	5TM-5TMR_LYT		26	0.036
Beta-propeller	Extracellular binding	Reg_prop		38	0.052
GBD	Galactose binding	7TMR-DISMED2		10	0.014
		CBM_4_9		2	0.003
			Subtotal:	*12*	*0.017*
TPR	Protein-Protein interactions	TPR_19		6	0.008

		ANAPC3		1	0.001
			Subtotal:	*7*	*0.010*
Cupin	Nucleotide binding	cNMP_binding		7	0.010
HHH	Helix-turn-Helix	HHH_5		2	0.003
E-set	Sugar binding	Y_Y_Y		10	0.014
Phosphatase	Cyanide binding	Rhodanese		10	0.014
P-loop-NTPase	Nucleotide binding	ABC_tran		1	0.001
		ABC_sub_bind		1	0.001
			Subtotal:	*2*	*0.003*
No assigned superfamily	Zinc binding	CZB		1629	2.248
	PilJ	PilJ		1095	1.511
	Nitrate and nitrite binding	NIT		923	1.273
	Oxygen binding	Hemerythrin		276	0.381
		PocR		166	0.229
	c-di-GMPbinding	PilZ		270	0.373
	Amino acid binding	FIST_C		220	0.304
		FIST		219	0.302
	Domain of unknown function	DUF3365		213	0.294
	Hydrogen binding	Fe_hyd_lg_C		124	0.171
	Adenosyl group binding	CBS		65	0.090
	Ammonium transporter	Ammonium_transp		64	0.088
	Integral membrane sensor	MHYT		36	0.050
	Ligand binding	PrpR_N		18	0.025
	Putative LBD	He_PIG		8	0.011
	Putative LBD	Response_reg		7	0.010
	Putative LBD	Htr2		7	0.010
	Domain of unknown function	DUF4077		5	0.007
	Putative LBD	AhpC-TSA		5	0.007
	Putative LBD	Pro_CA		4	0.006
	Integral membrane domain	MASE3		4	0.006
	Putative LBD	DapB_N		4	0.006
	Putative LBD	Pyr_redox_2		3	0.004
	Putative LBD	NADH_4Fe-4S		3	0.004
	Putative LBD	H_kinase_N		3	0.004
	Domain of unknown function	DUF2222		3	0.004
	Putative LBD	TPR_16		2	0.003
	Putative LBD	T7SS_ESX_EspC		2	0.003
	Phosphate/sugar binding	PTS_EIIC		2	0.003
	Putative LBD	MASE4		2	0.003
	Putative LBD	GGDEF		2	0.003
	Domain of unknown function	DUF948		2	0.003
	Putative LBD	B12-binding		2	0.003
	Putative LBD	AIP3		2	0.003
	Putative LBD	YkyA		1	0.001
	Putative LBD	Vps53_N		1	0.001
	Putative LBD	VPS28		1	0.001
	Putative LBD	Usher		1	0.001
	Putative LBD	UPF0184		1	0.001
	Putative LBD	Ufd2P_core		1	0.001
	Putative LBD	Transgly		1	0.001
	Putative LBD	Tox-URI2		1	0.001
	Putative LBD	TnpV		1	0.001
	Putative LBD	THF_DHG_CYH_C		1	0.001
	Putative LBD	THF_DHG_CYH		1	0.001
	Putative LBD	SURF1		1	0.001
	Putative LBD	SpoIIAA-like		1	0.001
	Putative LBD	SOR_SNZ		1	0.001
	Putative LBD	Sensor_TM1		1	0.001
	Putative LBD	SDH_alpha		1	0.001
	Putative LBD	Porin_4		1	0.001
	Putative LBD	PhoU		1	0.001
	Putative LBD	Pentapeptide_4		1	0.001
	Putative LBD	Pentapeptide		1	0.001
	Putative LBD	PapC_N		1	0.001
	Putative LBD	OMS28_porin		1	0.001
	Putative LBD	NAD_binding_3		1	0.001
	Putative LBD	Mut7-C		1	0.001
	Putative LBD	MerR_1		1	0.001
	Putative LBD	LXG		1	0.001
	Putative LBD	Lipoprotein_7		1	0.001
	Putative LBD	Ion_trans_2		1	0.001
	Putative LBD	ING		1	0.001
	Putative LBD	IGPD		1	0.001
	Putative LBD	IclR		1	0.001
	Putative LBD	HTH_ParB		1	0.001
	Putative LBD	Glyco_hydro_2_N		1	0.001
	Putative LBD	Glyco_hydro_106		1	0.001
	Putative LBD	FRB_dom		1	0.001
	Putative LBD	FHIPEP		1	0.001
	Putative LBD	Fe-ADH		1	0.001
	Putative LBD	F420_oxidored		1	0.001
	Putative LBD	Exonuc_VII_L		1	0.001

Putative LBD	EMC3_TMCO1	1	0.001
Putative LBD	EF-hand_11	1	0.001
Putative LBD	EAL	1	0.001
Domain of unknown function	DUF442	1	0.001
Domain of unknown function	DUF4118	1	0.001
Domain of unknown function	DUF3235	1	0.001
Domain of unknown function	DUF1640	1	0.001
Putative LBD	DNA_pol_A_exo1	1	0.001
Putative LBD	CHASE	1	0.001
Putative LBD	Cas_DxTHG	1	0.001
Putative LBD	bZIP_2	1	0.001
Putative LBD	BLUF	1	0.001
Putative LBD	Band_7	1	0.001
Putative LBD	Bac_rhodopsin	1	0.001
Putative LBD	Asparaginase	1	0.001
Putative LBD	Alanine_zipper	1	0.001
Quorum sensing	AI-2E_transport	1	0.001
Putative LBD	5_3_exonuc_N	1	0.001
Putative LBD	5_3_exonuc	1	0.001
Putative LBD	2CSK_N	1	0.001
	Subtotal:	*5449*	*7.52*
	TOTAL:	72480	100
