# Supplementary figures and images for: Prevalence and Specificity of Chemoreceptor Profiles in Plant-Associated Bacteria
Source: mSystems. 2021 Sep 21;6(5):e00951-21. doi: 10.1128/mSystems.00951-21 (PMC8547431; doi:10.1128/mSystems.00951-21)

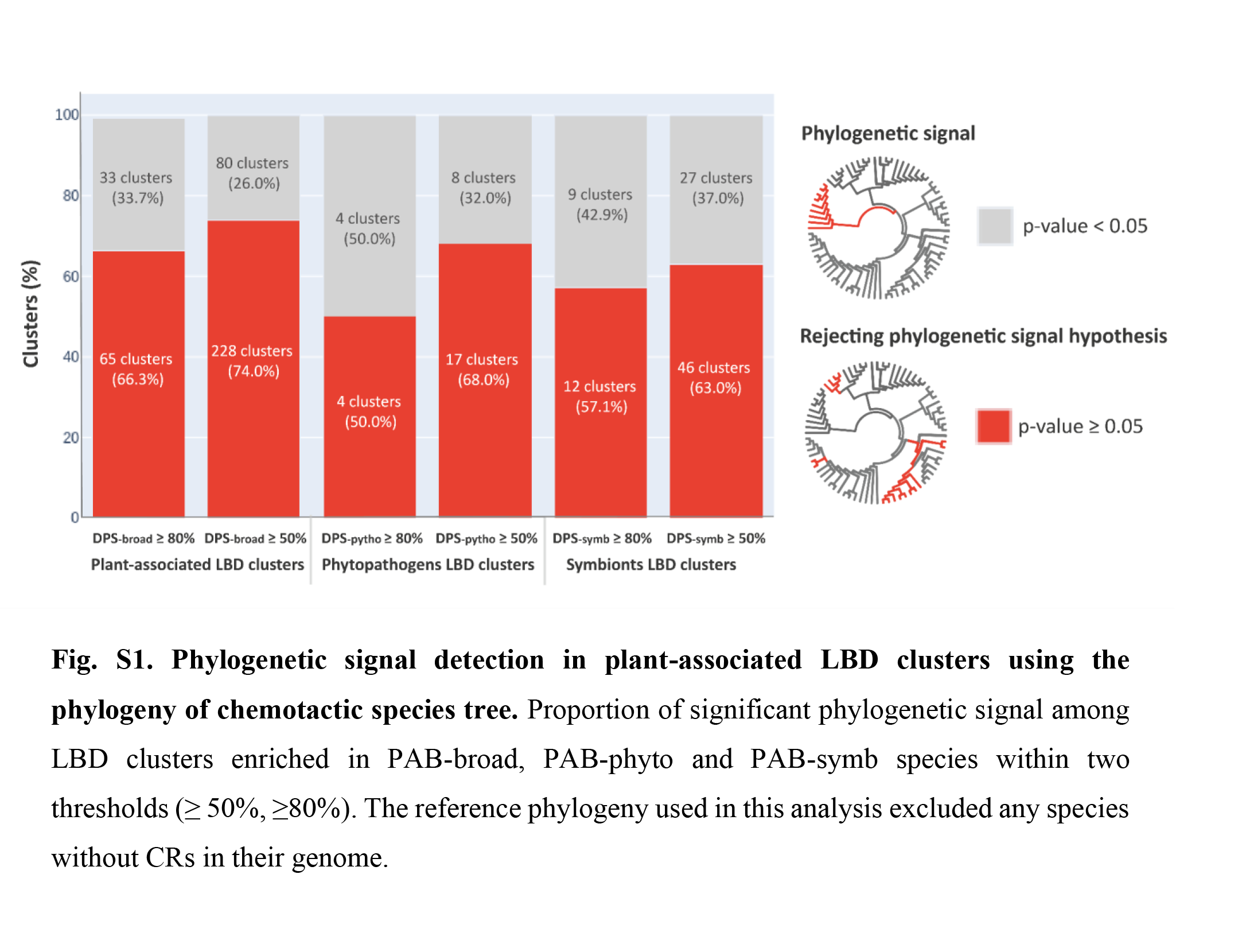

Supplement: FIG S1 [file msystems.00951-21-sf001.tif]
